# “Never Just the Next Case File”: A Qualitative Study Exploring Youth and Family Experiences Within Early Psychosis Coordinated Specialty Care

**DOI:** 10.1111/eip.70168

**Published:** 2026-03-26

**Authors:** Rabia Zaheer, Brooke Magel, Quincy Vaz, Nicole Kozloff, Augustina Ampofo, Lillian Duda, Janet Durbin, George Foussias, Sanjeev Sockalingam, Juveria Zaheer, Aristotle Voineskos, Sophie Soklaridis

**Affiliations:** ^1^ Department of Education Centre for Addiction and Mental Health Toronto Ontario Canada; ^2^ Department of Psychiatry, Temerty Faculty of Medicine University of Toronto Toronto Ontario Canada; ^3^ Campbell Family Mental Health Research Institute Centre for Addiction and Mental Health Toronto Ontario Canada; ^4^ Slaight Family Centre for Youth in Transition Centre for Addiction and Mental Health Toronto Ontario Canada; ^5^ The Wilson Centre University Health Network/University of Toronto Toronto Ontario Canada; ^6^ Institute for Mental Health Policy Research Centre for Addiction and Mental Health Toronto Ontario Canada; ^7^ Department of Family and Community Medicine, Temerty Faculty of Medicine University of Toronto Toronto Ontario Canada

**Keywords:** Canada, mental health, NAVIGATE, person‐centred, recovery

## Abstract

**Introduction:**

Early intervention is critical in preventing relapse and promoting recovery for people with psychosis; however, recovery‐based services are not always delivered consistently across early psychosis intervention programs. NAVIGATE, a coordinated specialty care program, was developed to standardise care for psychosis while embracing principles of person‐centred care. It has demonstrated promising recovery outcomes internationally; however, the experiences of youth with psychosis and their families in this program are less documented and more broadly are often neglected in research and mental healthcare. This study explored youth and family member experiences of the NAVIGATE program as part of a broader multi‐site implementation study in Ontario, Canada.

**Methods:**

Twenty‐two qualitative semi‐structured interviews with youth and family members were conducted using an open exploration approach to encourage participants to centre their experience in the program. Interview transcripts were analysed using thematic analysis.

**Results:**

Two key themes related to the impactful aspects of NAVIGATE were identified: (1) being treated as a person; (2) meaningful inclusion of family members. Overall, both youth and family members expressed a strong sense of satisfaction with the program. This satisfaction likely reflects the program's focus on shared decision‐making, individualization, and comprehensive care.

**Conclusion:**

Youth and family member perspectives are critical to inform the implementation and delivery of psychosis care that helps youth achieve their recovery goals and supports family members.

## 1 Introduction

Psychosis can be a frightening and perplexing experience (Fusar‐Poli et al. 2022; Noiriel et al. 2020). Direct accounts describe it as a whole human experience that may include voices, visions, messages, and alternative realities (Jones and Shattell 2016). Psychosis usually begins during adolescence or early adulthood (Calabrese and Al Khalili 2022). It not only affects the person who experiences it, but can also place immense caregiving responsibilities on family members (Oluwoye et al. 2020). Added to these challenges, youth are often confronted with a complex mental health care system that has limited resources, making it difficult to access early psychosis services (Iyer et al. 2015; Mueser et al. 2015).

Early psychosis intervention (EPI) programs were developed in response to evidence suggesting that early intervention is critical to prevent relapse and enhance recovery (Iyer et al. 2015). Specialised EPI programs result in better outcomes compared with standard treatment (Correll et al. 2018; Noiriel et al. 2020). Despite this, EPI programs have been criticised for lacking consistent implementation and standardisation in clinical practice, particularly in the delivery of recovery‐based services (Durbin et al. 2016; Kozloff et al. 2020). These shortcomings may explain why recovery rates remain low, despite the benefits of such programs (Lappin et al. 2018).

The NAVIGATE program was developed to standardise treatment for early psychosis, including evidence‐based psychosocial approaches (Mueser et al. 2015). This coordinated specialty care program features four components: (1) individualised medication management; (2) individual resiliency training; (3) supported employment and education; and (4) family education. Other services, such as peer support, may also be offered (Mueser et al. 2015). NAVIGATE has demonstrated positive outcomes such as improved quality of life and higher treatment retention rates in the US (Kane et al. 2016) and internationally (Roe et al. 2021), but little is known about its application in Canada. To address this gap, a broader implementation study titled “Early Psychosis Intervention: Spreading Evidence‐based Treatment” (EPI‐SET) assessed the impact of the NAVIGATE program on adherence to EPI standards of care and youth mental health outcomes across six sites in Ontario, Canada (Kozloff et al. 2020).

NAVIGATE embraces principles of person‐centred care, which challenge traditional medical approaches to recovery (Mueser et al. 2015). Despite the benefits of person‐centred care, it is difficult to implement in practice (Lodge et al. 2017; Stanhope et al. 2021), especially for youth with psychosis (Stanhope et al. 2021). Moreover, the implementation of mental health programs continues to face a broader conceptual tension: standardised, manualized programs such as NAVIGATE often conflict with the individualised focus that is central to person‐centred care (Garfield 1996; Hucker and McCabe 2012; Kvæl et al. 2022; Lee et al. 2023; Mannion and Exworthy 2017; Marshall 2009).

Determining the impact of the NAVIGATE program requires examining youth and family member experiences. Historically, youth and family voices have been neglected in mental health (Allerby et al. 2020, 2022; Boydell et al. 2010; Soklaridis et al. 2019). Research on early psychosis often prioritises quantitative methods to evaluate implementation and outcomes, while disregarding the perspectives of youth and their families (Boydell et al. 2010). The exclusion of youth and family voices is a significant omission because youth and healthcare teams do not always share the same goals (Allerby et al. 2020), and may have different perspectives on service engagement in EPI programs (Lal and Malla 2015). Research incorporating youth and family perspectives can illuminate meaningful elements in treatment and recovery (Malla et al. 2017). Specifically, qualitative approaches can be a useful way to explore the experiences of youth and their families who are involved in complex interventions such as NAVIGATE (Lewin et al. 2009; Reznik et al. 2025). Given this context, the current qualitative study aimed to explore how youth and their family members experienced the NAVIGATE program in Ontario, Canada.

## 2 Methods

### 2.1 Design

This study used a qualitative descriptive design to allow for in‐depth exploration of participant experiences (Doyle et al. 2020). In line with the constructivist paradigm, authors believe in the multiplicity of truths and that knowledge is co‐constructed between the researchers and participants. The authorship team is composed of researchers, psychiatrists, and people with lived experience. To minimise bias, the authors (RZ, BM, QV, SS) who conducted the interviews, analysis, and/or initial interpretation of the data were not involved in the implementation of NAVIGATE. These authors also regularly reflected on how their assumptions, preferences, and biases influenced the analysis and findings in regular weekly meetings. Notably, multiple phases of this research involved consultation or co‐creation with the Family and Youth Advisory Committees, including co‐creating the interview guides, consulting on analysis strategy and initial thematic development, and serving as authors or contributors on the manuscript.

### 2.2 Recruitment and Sample

This study recruited two youth (age 14–35) and two family members from each of the six sites. To be eligible, participants had to be proficient in English and have received services in the NAVIGATE program for at least 1 year. Purposeful sampling was employed to ensure that participants had sufficient and comprehensive experience with the intervention, and participants were also recruited based on convenience (Palinkas et al. 2015). Most youth were approached at a follow‐up visit for the broader EPI‐SET study or via telephone, email, or text message; family members were approached via telephone or email. Youth were asked to identify family members involved in their care. In our sample, this included parents, siblings, and partners. Youth and family members were not intentionally matched or paired. The final sample size was based on the principles of information power (Malterud et al. 2016), which refers to the level of relevant information contained in a sample based on the study aim, sample specificity, existing theory, interview dialogue quality, and analytic approach.

### 2.3 Data Collection

One‐on‐one semi‐structured interviews were conducted by authors (RZ, BM) over Webex video‐conferencing technology. Written informed consent was obtained using the REDCap e‐consent framework (Harris et al. 2009, 2019). The interview guide, informed by a template from Tsang (2008, personal communication) (see Data S1), was designed with an open exploration approach to encourage participants to centre their experiences and discuss what is most important to them in the NAVIGATE program. Participants led the discussion, and interviewers prompted them with open‐ended, exploratory questions. Interviews were audio‐recorded, and a transcriptionist provided verbatim transcription. The interviews occurred between August 2022 and February 2023, and lasted 33–60 min. Participants received a $30 e‐gift card honorarium.

### 2.4 Data Analysis

The study followed Braun and Clarke's six‐step thematic analysis approach (Braun and Clarke 2006). First, authors familiarised themselves with the data, which involved several rounds of reading three randomly chosen transcripts. Then, they generated initial codes using an inductive approach to coding and began identifying and labelling meaningful excerpts in the transcripts. Three authors (BM, QV, RZ) met to share their interpretations of codes generated from the transcripts to create an initial codebook. One author (QV) used the codebook to code the remaining transcripts on Dedoose (Dedoose 2023). During the coding process, authors met regularly to refine the codebook to ensure consistency and enhance trustworthiness. Then, they performed “code chunking”, where similar codes were grouped to identify patterns and generate initial themes. They actively pursued contrasting and unique participant perspectives (Cohen and Crabtree 2006) to highlight diverging viewpoints, which allowed for more nuanced perspectives. Next, the themes were refined to produce subthemes and the interpretations were discussed with the Family and Youth Advisory Committees as a form of external member checking. Lastly, they collectively engaged in a reflexive process, where they carefully considered each theme and assigned a name to it.

## 3 Results

### 3.1 Demographics

Twenty‐two participants (11 youth and 11 family members) from six sites participated in the interviews. Given the limited number of eligible youth at one site, eligibility criteria were expanded to include one participant who had received NAVIGATE services for less than 1 year. At the time of the interviews, *n* = 4 (36.4%) youth were currently receiving the NAVIGATE intervention. The remaining youth were discharged from the program between 3 and 24 months (*M* = 11, SD = 6.8) (Table 1).

**TABLE 1:** Sample demographic characteristics.

Youth demographic characteristic (*n* = 11)	
Gender *n* (%)	
Man	6 (54.5)
Non‐binary	1 (9.1)
Woman	4 (36.4)
Age	
Mean (SD)	25.3 (4.35)
Median	25
Range	19–32
Race^a^ *n* (%)	
Black African	1 (9.1)
South African	1 (9.1)
South Asian	1 (9.1)
White	5 (45.4)
Prefer not to answer	3 (27.3)

Family demographic characteristic (*n* = 8)^b^	
Gender	*n* (%)
Man	3 (37.5)
Woman	5 (62.5)
Age	
19–25	1 (12.5)
26–34	1 (12.5)
35–44	1 (12.5)
45–54	2 (25)
55–64	3 (37.5)
Race^a^	
First nations	1 (12.5)
South Asian	1 (12.5)
White	6 (75)
Relation to youth	
Sibling	1 (12.5)
Spouse/Partner	2 (25)
Parent	5 (62.5)

^a^ Self‐identified.

^b^ *n* = 3 missing.

Youth and family members expressed a strong sense of satisfaction with the NAVIGATE program. All participants indicated they would recommend the program to someone experiencing early psychosis. Two themes were identified that relate to the impactful aspects of NAVIGATE: (1) being treated as a person; (2) meaningful inclusion of family members.

### 3.2 Theme 1: Being Treated as a Person

Participants felt that the program respected their personhood and actively incorporated their opinions, interests, and lifestyles into treatment. The program also recognized youths' employment and education goals as central to recovery. Both youth and family members reminisced on celebratory milestone moments with the NAVIGATE team, such as birthdays and graduations.

#### 3.2.1 “[My NAVIGATE team] let me lead, let me guide”

Both youth and family members described how the NAVIGATE team valued youth and respected their agency. Family members mentioned that the team listened to the youth, treated the youth with dignity, and did not “look at them differently” because of their psychosis. This suggests that their welcoming and non‐judgmental attitude created an environment for open dialogue.

“The staff at the program were knowledgeable, supportive, not intimidating, and they're welcoming. I think that is one of the bigger takeaways. You don't feel as if things are imposed on you. You don't feel stupid for asking a question. It's just an overall well‐organised support system.”—Family member 07

Many participants appreciated co‐leading care and sharing decision‐making with the NAVIGATE team.

“I also still liked the overall approach that everyone had. It wasn't someone telling me what to do […] I had a full seat at the table when it came to my care and talking about what I felt I needed.”—Youth 01

It appears that this collaboration allowed the team to make the treatment “unique” based on the youth's needs and desired goals.

“For every patient it's different […] They're asking him [about his preferences] so that they can find [what could work] for him.”—Family member 06

Some participants felt that the ability to make shared decisions and have a choice in their treatment, in turn, instilled a sense of independence and self‐confidence in some youth and in some cases improved self‐esteem.

“Confidence. I think she gained independence. I think she gained a sense of being in control of her own path forward. You could see it grow over time.”—Family member 04

#### 3.2.2 “Only medicine cannot do”

Participants felt that having an interdisciplinary team with specialised EPI training gave them access to all the resources and support they needed in one place.

“It was good that it was a whole team. It wasn't just like a doctor or a nurse. It was a doctor, a nurse, an OT, and social worker.”—Youth 10

“You're not just seeing a psychiatrist who administers meds and has 15 minutes for you. You're not just seeing a counsellor who can't adjust your meds and can only talk to you, because what if you need medication? You have a nurse who is checking in on you and making sure that you're getting bloodwork. That's a full approach.”—Youth 01

Participants indicated that NAVIGATE incorporated their preferences and lifestyle in their recovery journey and provided opportunities to engage in activities that were fulfilling to them. For example, for some participants, the most memorable components of the program included doing art or going out for a snack with team members or peers in the program.

“For my caseworker, it was redirecting my energy from self‐harm to ‘You like tattoos—how about get a tattoo?’ or ‘You like art—let's go and do some art and put your emotions into something more productive.’ That helped […] redirecting my energy to working out or getting a coffee […] They organised a bowling day. It was cool.”—Youth 06

“He meets for coffee [with a team member]! It makes his day. It makes him smile again.”—Family member 06

Many youth voiced that the NAVIGATE team empowered them to pursue their educational and career goals. This included providing support with accommodation letters, doctor's notes, and career development resources. They attributed the program to helping them meet key developmental milestones.

“If I'm having a problem at work, they are keeping me safe and providing me with support. I did not like my job, so I told [the supported employment and education specialist] that I wanted to change […] He talked to the manager and they put me in a [different position].”—Youth 05

“It's almost like going through a car crash and rehabilitating after, but it was a mental car crash and rehabilitating after. I finished my degree […] I started working. I have healthier, stronger, improved relationships. I think that they facilitated most of those abilities.”—Youth 07

#### 3.2.3 “We did not have to go hunting or looking for programs”

The majority of the participants were grateful to be connected to NAVIGATE in a streamlined process with short waitlists, smooth referrals, and discharge. Some attributed the program to preventing further hospital visits and deterioration of youths' mental health.

“I got out [of the hospital], and I came out with a team on my side, and a full stream of medication for almost a year […] That was free. That's a pretty good thing to have in a society.”—Youth 07

Some participants offered feedback such as increasing the duration of the program, and/or providing follow‐up appointments after discharge. While many participants appreciated that they could reach out to the NAVIGATE team whenever they needed, a few felt that offering appointments outside traditional work hours could make EPI care and support even more accessible.

“I just think it would be nice if they were connected after [the end of the program] somehow in a stronger form. Maybe it's once every six months that they check in.”—Family member 04

#### 3.2.4 “She was there not as a care provider but as someone who did care”

Some participants felt that the NAVIGATE team was shifting the boundaries of care, moving beyond traditional patient‐provider relationships to playing an active role in youths' lives.

“One of my team members made the trip to the hospital to come and sit with me and spend some time with me [on my birthday] […] she made the active effort to take time off of work, drive all the way to see me so she could support me.”—Youth 11

“When he [completed the program], they had cake […] They made him feel that they were happy that they worked with him.”—Family member 05

They indicated that NAVIGATE team members were “genuinely interested in their lives” and encouraged them to pursue their passions and hobbies.

“They're not just interested in the medication or the medical side of it. They are interested in you as a person.”—Youth 02

“I do 3D printing, and that's typically how our appointments with my psychiatrist start. She asks what I've been printing […] There have been a couple of points where things just weren't interesting anymore. But the program has helped me get back into them, and I don't think I've lost any passions or hobbies since.”—Youth 02

Several participants expressed that during the COVID‐19 pandemic, the NAVIGATE team went to great lengths to minimise treatment disruptions, for example, by travelling to youths' homes to administer medication. For some participants, this dedication made the NAVIGATE team akin to neighbours or family.

“They just seemed like your neighbours […] They were just friendly, but it also felt personal. That doesn't happen often.”—Youth 03

“I feel like [the team] is also a part of my family, or more than that, because they did more than what a family can do.”—Family member 03

### 3.3 Theme 2: Meaningful Inclusion of Family Members

Most participants valued the emphasis the NAVIGATE program places on family engagement. Family participants described playing an active role in the youth's recovery and receiving support themselves through family education.

#### 3.3.1 “Not left behind”

Participants emphasised the importance of family involvement in the NAVIGATE program, describing family members as advocates for the youth, who provided emotional support and helped with scheduling, transportation, and attending appointments. Families were also a source of motivation and accountability.

“My dad was a big support. I think he went through it with me from the beginning, even before I started in recovery. He just stuck with it the whole time. He was the biggest support through the whole thing. He was connected with [the NAVIGATE team]. They have a family nurse, so I think he was connected through that.”—Youth 03

Family members appreciated how the program incorporated their perspectives and feedback and engaged them in a meaningful way.

“It does make me feel that the care is a little bit more complete than a lot of other standard care, where most of the time the doctors won't reach out to family members unless you specifically ask them to.”—Family member 10

They described the stress, fear, and anxiety they experienced as caregivers and how the program supported them throughout the youth's journey to recovery and helped them bear their responsibilities as caregivers.

“They guided us step by step […] They took care, so at least we were not alone and we were not in limbo.”—Family member 03

“They never made any of us feel like we were just the next case file or anything like that. There was a really good human connection there.”—Family member 07

#### 3.3.2 “A learning experience”

Many family members valued the psychoeducation component of the program. At some sites, the program offered group‐based family education (courses and webinars) or family peer support groups. Some participants emphasised how it was helpful to learn about crisis intervention and tertiary prevention (identifying warning signs of relapse or adverse effects of long‐term medication).

“We did the [family education] course twice because we were so overwhelmed […] There was so much helpful information in understanding the diagnosis and understanding how to support and help your loved one, and medication.”—Family member 11

They indicated that this psychoeducation added to their lived family experience and transformed their understanding of psychosis.

“It's partly a relearning as well, because there were some issues that I was not trained on as a [health care worker].”—Family member 02

## 4 Discussion

To our knowledge, this is the first qualitative study to explore the perspectives of youth and family members in the NAVIGATE program in Canada. This study found that participants had very positive experiences with the program and that these experiences seemed to be grounded in the program's focus on person‐centred care (Santana et al. 2018), such as shared decision‐making, individualised, and comprehensive care. Similar to our findings, literature has linked person‐centred care to improvements in self‐esteem, confidence, program engagement and recovery (Allerby et al. 2022). It was encouraging to find that most youth felt connected with their care team, appreciating the compassion and care that were demonstrated, which are traits highly valued by people receiving care (Coulter and Oldham 2016). Our findings align with qualitative research in the US on the importance of patient‐provider relationships in EPI interventions. For example, participants in Daley et al.'s (2020) study emphasised the warm and empathetic demeanour of their providers. Similarly, van Schalkwyk et al.'s (2015) study exploring patient experiences found that participants highly valued trusting relationships with their therapist, specifically noting that they felt heard. These studies also reinforce the value of integrating employment, education, and housing services into EPI services. Participant experiences in NAVIGATE are particularly noteworthy because manualized treatment programs have been criticised for lacking flexibility and being unable to offer individualised person‐centred care (Galovski et al. 2024; Truijens et al. 2019). Our findings suggest that it is possible to provide standardised treatment for early psychosis while tailoring care to meet individual needs.

The experience of families within coordinated specialty care for early psychosis has not been widely documented (Lucksted et al. 2018). Family members in our study reported regular communication, collaboration, and opportunities for psychoeducation and support from other family members in the NAVIGATE program. These findings should be interpreted within the context of the broader implementation study (Barwick et al. 2025), which noted that adherence to fidelity for the Family Education and Support component of NAVIGATE changed from a rating of poor to fair a year after implementation. While the fidelity results are promising, the authors noted that further improvements are needed to meet the Ontario EPI Program Standards (Ministry of Health and Long‐Term Care 2011).

Family members' positive experiences in this program stand in contrast to findings in a descriptive review of qualitative studies, which show only minimal or informal family involvement, leading families to feel invisible or undervalued in psychosis treatment programs (Boydell et al. 2010). Other literature reports mixed experiences, as Mui et al.'s (2019) systematic review of qualitative studies found that some caregivers perceived EPI services to be helpful while others felt undervalued. Similarly, a long‐term follow‐up qualitative study in Norway found that caregivers were rarely included in treatment, albeit instrumental for psychosis recovery (Åsbø et al. 2025). This experience extends to mental health care and research in general (Rodolico et al. 2022; Soklaridis et al. 2019; Velleman et al. 2005) and is concerning because families are often informal caregivers and experience significant economic and social burden (Moudatsou et al. 2021). As suggested by our participants and the broader literature (Doyle et al. 2014; Onwumere et al. 2018; Hansen et al. 2018), family members have the potential to exert a positive influence on treatment outcomes by being a source of motivation and accountability. Family involvement in NAVIGATE may have been successful because the program emphasises shared decision‐making and encourages active involvement of families in care (Drapalski et al. 2018; Eassom et al. 2014). Continued engagement is crucial to ensure meaningful family involvement in EPI programs.

Although our study participants reported quick and easy access to the NAVIGATE program, there are many inequities in accessing early psychosis care. For example, the majority of EPI services in Canada are located in urban areas and are sparse in rural and remote communities (Iyer et al. 2015). Further, research from the US (Oluwoye et al. 2021) and Canada (Polillo et al. 2023) examining pathways to treatment for early psychosis demonstrated racial and ethnic disparities in access. It is also important to note that while participants generally agreed that the program discharged youth at the appropriate point in their recovery, a couple of participants wished the program were longer. Based on the Ontario EPI Program Standards, EPI services in the province are delivered for up to 3 years (Ministry of Health and Long‐Term Care 2011). Determining the optimal duration of EPI services and identifying ways to maintain treatment gains after discharge, including the potential benefits of extending EPI services (Bertelsen et al. 2008; Lenior et al. 2001; Norman et al. 2011), should be explored further.

Our findings have important clinical implications. First, supporting the youth to identify and set goals related to their care is a key task of EPI services. Second, family members should be proactively involved in the youth's care, as they are often the caregivers and invisible experts (Abou Seif et al. 2022). Third, EPI care should include an interdisciplinary team to not only address clinical symptoms through medication management, but also attend to the social and psychological factors that can be crucial for high treatment engagement and positive recovery outcomes. In particular, many participants in our study highly valued the supported employment and education in NAVIGATE. Lastly, as shown in the literature (Polillo et al. 2022) and highlighted by our participants, flexibility can improve engagement in EPI services. In our study, this includes both how, when, and where the care is delivered (e.g., virtual appointments and availability outside of business hours).

## 5 Limitations

Participants had some difficulty recalling specific aspects of their experiences (e.g., NAVIGATE modules). This difficulty may have been due to the significant amount of time that had passed since leaving the program or, in the case of youth, the nature of their psychosis. However, participants were able to remember their overall experience. Most participants had an overwhelmingly positive experience with NAVIGATE, perhaps because of their limited experience with other EPI services. These positive experiences may also reflect the gratitude of receiving any support in a system with significant barriers to care, including long wait times, high costs for services outside the public system, and geographic inequities (Iyer et al. 2015). Alternatively, this may be a result of attrition bias, in which participants with less favourable perspectives may have left the program early and thus did not meet our one‐year inclusion criteria. Our participant group likely reflects a more satisfied subset of youth and family members remaining in the program for over a year. A strength of this study is that it illuminated factors that contribute to long‐term program engagement; however, future research should explore reasons for early disengagement in EPI. Lastly, most participants received care during the COVID‐19 pandemic, which required changes to program delivery (e.g., virtual appointments and modified/cancelled activities).

## 6 Conclusion

Our study contributes to the expanding literature on centring the perspectives of youth and families in EPI services and research. Providing EPI programming that is both holistic and person‐centred is important to youth and families seeking care. Efforts aimed at improving the quality and impact of EPI services should establish mechanisms that determine whether youth and families are experiencing the program in these ways, with the ultimate aim of increasing program engagement and effectiveness.

## Funding

This work was supported by the SPOR Innovative Clinical Trial Multi‐Year Grant, Canadian Institutes of Health Research (347737).

## Ethics Statement

Research Ethics Board approval was obtained from the Centre for Addiction and Mental Health, which was the coordinating site for the study, and all six study sites (Canadian Mental Health Association Thunder Bay, Canadian Mental Health Association Waterloo Wellington, Health Sciences North, Lakeridge Health, Niagara Region Public Health, and North Bay Regional Health Centre). All study procedures were conducted per national (TCPS2, 2022) and international ethical guidelines (Declaration of Helsinki and Nuremberg Code).

## Consent

Written informed consent was obtained from participants for publication during the consent procedure.

## Conflicts of Interest

The authors declare no conflicts of interest.

## Supporting information


**Data S1:** Qualitative Interview Guide.

## Data Availability

Research data are not shared.
