# Supplementary material for: “Never Just the Next Case File”: A Qualitative Study Exploring Youth and Family Experiences Within Early Psychosis Coordinated Specialty Care
Source: Early Interv Psychiatry. 2026 Mar 26;20(4):e70168. doi: 10.1111/eip.70168 (PMC13021323; doi:10.1111/eip.70168)
Supplement: Supplementary file 1 — Data S1: Qualitative Interview Guide. [file EIP-20-0-s001.docx]

## Qualitative Interview Guide - FAMILY VERSION

This interview guide was adapted from a template created by Dr. A. Ka Tat Tsang (Tsang, 2008).

Thank you for giving us the time to do this interview with you. The main purpose of this interview is to understand your thoughts and feelings about your family member’s care in the NAVIGATE program.

| Interview Topics | Sample Interview Questions |
| --- | --- |
| Ice Breaker | 1. **I recognize that we are all so busy with our time, was there something that drew you into joining this research project?**    1. Can you expand on that? 2. **Tell me what interested you about this project?**    - Why? Can you expand? |
| Joining NAVIGATE | 1. **How did your loved one join the NAVIGATE program?**    1. How did you find out about the program?    2. How did you get into it?    3. How did it make you feel? 2. **Have you tried a different program before joining NAVIGATE?**    1. If yes, what was it like? 3. **Did you have any expectations before joining the NAVIGATE program?**     1. What were they?    2. Have your expectations been met? |
| Having a loved one in the program | 1. **What was it like having your loved one in the program?**    1. How did you feel about it?       1. Happy/Sad/Angry/Confused? |
| Resources | 1. **Did the program provide any education for family members?**    1. Was the information helpful?    2. What wasn’t helpful?    3. Did you get all the information you needed?    4. Did you learn anything about your loved one’s journey?    5. Did you learn anything about your loved one’s diagnosis? 2. **Did you receive any resources for your loved one?**    1. What were those resources?    2. Did you find those resources useful?    3. Were you directed to other resources? (i.e., meso, macro, micro level resources)    4. Did you learn anything about your loved one’s diagnosis from these resources? 3. **Does NAVIGATE offer services to family members [such as yourself]?**    1. Have you used these services? Why/Why not?    2. Do you attend these services? Why/Why not? |
| Support | 1. **Did you feel supported in the program? (i.e., by staff, other family members, etc.)?**    1. What did that support look like?    2. What did that support not look like?    3. How did that support feel?    4. Did you feel supported navigating your loved one’s journey? |
| Involvement in Care | 1. **What was your experience being involved in your loved one’s care?**    1. What was difficult about being involved?    2. What was hard about being involved? 2. **Could you tell me about your family’s investment into this program to ensure that your loved one could participate in this program?** E.g., time, money, commitment, covid-19    1. What did it look like to take your loved one to the program? |
| Mindset | 1. **Have your views about illness and treatment changed overtime?**    1. What changes in your views have you noticed?    2. Have you noticed these views changing in any other family member? 2. **As you are learning about a loved one’s illness, what did your learning process look like?**    1. Do you think differently from when your loved one was first diagnosed to now? |
| Barriers | 1. **Have you experienced barriers during your loved one’s care?**    1. What were these barriers?    2. Did you experience frustration? Burn out? |
| Confidentiality | 1. **What do you know or don’t know about your involvement in the NAVIGATE program?** 2. **What do you know about confidentiality?** 3. **Did you ever experience a situation where you were balancing between your loved one’s autonomy and your own desires to be involved in their care?**    1. Did you ever experience conflicting priorities of care? |
| Ways of Knowing or Inclusion | 1. **Whose knowledge or ‘ways of knowing’ is represented in the NAVIGATE program?**    1. Was there a dominant mindset?    2. Whose knowledge was represented in the program?    3. Did you hear from people with lived experience? |
| Life Stages | 1. **What was it like transitioning through your loved one’s developmental life stages?**    1. E.g., College/University    2. E.g., Moving away/Moving home/Moving to a different city/town/country    3. Age progression, etc. |
| Changes to the program | 1. **In a perfect world, what would you change about this program?**    1. What did you like? What did you dislike?    2. Do you see yourself or your loved one reflected in the NAVIGATE program? |

**Follow up Questions:**

*Ways in which to ask to follow up questions about sensitizing topics (probes and clarification)*

1. Can you tell me more about that (person, event)?
2. Can you give me a specific example?
3. Can you explain your answer?
4. In what way?
5. How did you understand that?
6. What does that mean to you?

***Wrap up questions:***

1. Do you have anything to add?
2. Is there anything I should have asked?
3. How did the interview feel for you?
4. Is there anything that surprised you?
5. How are you feeling now?

## Qualitative Interview Guide – YOUTH VERSION

This interview guide was adapted from a template created by Dr. A. Ka Tat Tsang (Tsang, 2008).

Thank you for giving us the time to do this interview with you. The main purpose of this interview is to understand your thoughts and feelings about your experience with the NAVIGATE program. We are most interested in your personal experience. The purpose of this interview is to explore and discover.

| Interview Topics | Sample Interview Questions |
| --- | --- |
| Ice Breaker | 1. **Was there something that drew you into joining this research project?**    1. Can you expand on that? 2. **Tell me what interested you about this project?**    - Why? Can you expand? |
| Joining the NAVIGATE Program | 1. **How did you join the NAVIGATE program?**    1. How did you find out about the program?    2. How did you get into it? Who initiated your involvement?    3. How did you feel before you joined the program? 2. **Have you tried a different program before joining NAVIGATE?**    1. If yes, what was it like? 3. **Did you have any expectations before joining the NAVIGATE program?**     1. What were they?    2. Have your expectations been met? |
| Being in the Program | 1. **What was it like being in the program?**    1. How did you feel about it?       1. Happy/Sad/Angry/Confused? |
| Resources | 1. **Did the program provide you with any education?**    1. Was the information helpful?    2. What wasn’t helpful?    3. Did you get all the information you needed?    4. Did you the education teach you anything about journey? 2. **Did you receive any resources?**    1. What were those resources?    2. Did you find those resources useful?    3. Were you directed to other resources? (i.e., meso, macro, micro level resources)    4. Did you learn anything about your diagnosis from these resources? |
| Diagnoses | 1. **Do you agree with your diagnoses?**     1. Why or why not? 2. **Did you experience any barriers to getting a diagnosis?**     1. Probes: financial, time, transportation, trust, power dynamics    2. After diagnosis probes: internal struggles/personal barriers, anxiety, prejudice/stigma of having a diagnosis, disbelief diagnosis is real by self or family/friends. |
| Treatment | 1. **How was your treatment?**     1. What kind of treatment did you have?       1. Often asked to complete scales, if discussed, ask. What is the process like? What are your thoughts on the scales?    2. Did you participate in alternative forms of treatment?       1. E.g., dance, art, etc.    3. Only talk about medication if brought up. 2. **Did you learn about any coping strategies in NAVIGATE?**     1. What are they?    2. Do you think they are good or bad coping strategies? 3. **Were there any gaps or weaknesses in your treatment?**     1. Can you tell me about those?    2. What did or did not work for you?    3. Was your treatment plan right for you? 4. **Was it hard or easy understanding the information about your treatment or diagnosis?**     1. How did you ask for help?    2. What were some examples of things people did to help you understand?    3. What do you wish was done differently?    4. What would you like to teach them? 5. **Did you feel like an expert in your care?**     1. What was this like?    2. Was this expert knowledge respected? Acknowledged? |
| Support  (Feeling like a ‘number’) | 1. **Did you feel supported in the program by people in your life? (i.e., by staff, other family members, etc.)?**    1. What did that support look like?    2. What did that support not look like?    3. How did that support feel?    4. Are there other places that you turned to for support? |
| Involvement in Care  (Autonomy vs. dependence) | 1. **Who is involved in your care (family/friends/etc.)?**     1. Can you give me an example of a time it helped to have [insert person] involved in your care?    2. Can you give me an example of a time it didn’t help to have [insert person] involved in your care? |
| Mindset  (Negotiating illness/identity) | 1. **Have your views about illness and treatment changed overtime?**    1. What changes in your views have you noticed? 2. **As you are learning about your diagnosis, what did your learning process look like?**    1. Do you think differently now from when you were first diagnosed? 3. **What does a ‘good’ quality of life mean to you?**     1. Are there any barriers to achieving this? 4. **What are you excited about these days?** 5. **Do you have any passions in your life?**     1. Are you more/less or equally involved with them after starting treatment? |
| Equal Participation | 1. **What did it look like to ensure you could participate in this program?** E.g., time, money, commitment, covid-19    1. Were there any things that made it difficult to come to appointments? What would help with that? |
| Barriers | 1. **Have you experienced barriers during your care?**    1. What were these barriers?    2. Did you experience any strong emotions?       1. E.g., frustration? Burn out? Happiness? |
| Confidentiality | 1. **What do you know about confidentiality?** 2. **Did you ever experience a situation where you were balancing between your autonomy and your loved one’s own desires to be involved in your care?**    1. Did you ever experience conflicting priorities of care? What was it like? |
| Ways of Knowing or Inclusion | 1. **Whose knowledge or ‘ways of knowing’ is represented in the NAVIGATE program?**    1. Was there a dominant mindset or voice represented?    2. Whose knowledge was represented in the program?    3. Was lived experience incorporated into the model of care? |
| Life Stages | 1. **What was it like transitioning through your developmental life stages?**    1. E.g., College/University    2. E.g., Moving away/Moving home/Moving to a different city/town/country    3. Age progression, etc. |
| Resilience | 1. **In the context of your experience with this program, what does resilience mean to you?** |
| Changes to the program | 1. **In a perfect world, what would you change about this program?**    1. What did you like? What did you dislike?    2. Do you see yourself or your loved one reflected in the NAVIGATE program? 2. **If you had someone close to you experience psychosis, would you recommend them the program?**     1. Why or why not? |

***FOLLOW UP QUESTIONS:***

***Ways in which to ask follow up questions about sensitizing topics (probes and clarification)***

1. Can you tell me more about that (person, event)?
2. Can you give me a specific example?
3. Can you explain your answer?
4. In what way?
5. How did you understand that?
6. What does that mean to you?

***END OF INTERVIEW:***

***Wrap up questions***

1. Do you have anything to add?
2. Is there anything I should have asked?
3. How did the interview feel for you?
4. Is there anything that surprised you?
5. How are you feeling now?
